# Supplementary material for: Potential risk factors for cardiovascular diseases and associated sociodemographic characteristics: A cross-sectional evaluation of a large cohort of women living with HIV in north-central Nigeria
Source: PLOS Glob Public Health. 2023 Dec 5;3(12):e0002667. doi: 10.1371/journal.pgph.0002667 (PMC10697517; doi:10.1371/journal.pgph.0002667)
Supplement: Attachment — Submitted filename: Point by point response file_June 2023.docx [file pgph.0002667.s002.docx]

| **Section** | **Editor/Reviewers’ comments** | **Author’s response** |
| --- | --- | --- |
| **Reviewer #2** | | |
| General comments | This paper presents a description of the prevalence of three risk factors of cardiovascular diseases among women living with HIV in north-central Nigeria using data from a multi-center cross-sectional study. The association between several sociodemographic characteristics and the risk factors are also explored. I think the conclusion of the study can be substantially refined and some other changes and editing are needed.  Major issue:  Need overhaul of the conclusion, which is not quite related to the analysis. For example, the study didn’t investigate the effects of smoking and alcohol use on CVD risks; on the other hand, if it is true that smoking and alcohol use would increase the risks, the analysis is not needed to know the conclusion that targeting smokers and alcohol users would be useful. | Thanks. We agree we didn’t investigate the effects of smoking and alcohol use on CVD risks. We refrained from limiting our conclusion to smokers and alcohol users and stated the need to implement interventions that could foster lifestyle behavioral modification among women living with HIV on antiretroviral treatment.  See:  Abstract, conclusion, page 3, lines 50 - 51  Main manuscript, conclusion, page 16 line 278 |
|  | | |
| 1. | Title: the title is not quite accurate based on this study, because it doesn’t investigate the risk factors for CVD. | We meant “potential cardiovascular risk factors”. This has been corrected on the title page (see page 1, line) and in the manuscript. |
| 2. | Line 42 – 46, 166 - 172: it would be more informative to include the confidence intervals of the estimates. | This has been addressed. See pages 1 – 2, lines 42 – 48. |
| 3. | Line 57 – 59: more clarification is needed about why CVD has become a major health concern for PLHIV | Thanks. The chronic immune activation and inflammation associated with HIV and antiretroviral treatment contribute to cardiovascular atherosclerosis (Henning et al. 2023). We have included this for further clarification.  See page 4, lines 60 – 61. |
| 4. | Line 75: the research is not really about predicting the risk factors using the sociodemographic characteristics | We meant “potential cardiovascular risk factors and the association with sociodemographic characteristics”. The objective has been corrected. See page 4 lines 74 – 78. |
| 5. | Line 96: it would be better to use full name when the abbreviation appears the first time in paper. E.g., MTCT. | MTCT has been defined at first use. See page 5, line 97. |
| 6. | Line 80, 118: the number of participants is not consistent, and the difference is not explained. | The difference in the number of participants in the study and the final analyzed data was due to the removal of observations with missing information. This has been explained with a sentence. See page 6, lines 120 – 121. |
| 7. | Line 111, 121: marital status was collected but not described? | This has been added. See page 7, lines 142 – 143 |
| 8. | Line 121: why is the age dichotomized? What’s the rational for choosing 40 years as cutoff age? | Potential risk factors for CVD are age-related and 40 years cut-off has been used in prior studies cited in references 5 (Ogunmola et al 2014) and 6 (Desalu et al 2009). Therefore, age was dichotomized to enable comparability of our findings with earlier studies.  See page 7, lines 124 – 125. |
| 9. | Line 125: why is contraceptive use considered a cardiovascular risk factor? Further clarification would be helpful. | Oral contraceptives predispose to CVD by causing thrombosis. This explanation has been added. See page 7 line 129. |
| 10. | Line 127: what’s global null hypothesis test? Maybe need some citations and explanations. How could this be used to assess the fit of models? | A p-value of less than 0.05 for the global null hypothesis indicates model fit. The sentences have been revised to include “We assessed the fitness of each of our models using the global null hypothesis test. A p-value of less than 0.05 was considered statistically significant indicating the models were fit”. We have cited 2 papers (Kumbeni et al 2021, Apanga and Kumbeni 2021) for the global null hypothesis test approach for model fitting.  See page 7, lines 130 - 133 |
| 11. | Line 149: the number doesn’t seem to match the table. | Good catch! This has been corrected. Thanks. See page 9, line 153 |
| 12. | Table 3: the stars (*) are missing for some numbers. | Good catch! This has been added. Thanks. See page 11, Table line 184 |
| 13. | Line 188 – 191: it would be better to qualify the statements, since this is not a causal study | Another sentence has been added “The demographic characteristics were associated with the outcomes, namely, contraceptive use, alcohol consumption, and smoking but not causally related.  See page 12, lines 194 – 196. |
| 14. | Line 200: the citation is not consistent. | Good catch! This has been corrected. Thanks. See page 12, line 205 |
| 15. | Line 204: it would be helpful to have some evidence to support the argument. | Cited two references (Baum et al. 2010, Hahn et al. 2010) to support the potential for HIV disease progression with alcohol consumption. See page 12, line 210 |
| 16. | Line 212: it helps to clarify why alcohol assumption increases risky behavior; it could also be the other way around | Thanks. Alcohol consumption increases risky sexual behavior in individuals through disinhibitory mechanisms resulting in impaired judgment and increased likelihood of engaging in unprotected sex (sex with multiple partners and inconsistent use of condoms). We have edited the text for clarity and cited an additional reference (Bryant et al. 2010). See page 13, lines 214 – 216. |
| 17. | Line 271: it might not be reasonable to say that the prevalence is desirable since it is objective. | Agreed. Thanks. This has been corrected to “A low prevalence of smoking exists among women living with HIV on antiretroviral treatment” See page 16, line 276. |
| 18. | Need some editing: Line 214 – 217, 224, 233 – 236, 241 – 242, 248, etc. | The entire manuscript has been copy-edited. All grammar-related issues were addressed. |
| 19. | Grammar issues: line 92, 202, 209, 221, etc. |  |
| 20. | Other limitations not discussed: cross-sectional study might limit the inference made; the representativeness of data and selection bias; other major risk factors for CVD are not studied (hypertension, obesity, hypercholesterolemia) | We have updated the limitation section. We indicated “We were only able to study WLHIV in facilities where we received administrative approval for the study, and we acknowledge the potential for selection bias”. These limitations and our inability to study other major risk factors for CDV such as hypertension, obesity, and hypercholesterolemia have been stated.  See page 15, lines 263 – 266, 272 |
