# Supplementary material for: Potential risk factors for cardiovascular diseases and associated sociodemographic characteristics: A cross-sectional evaluation of a large cohort of women living with HIV in north-central Nigeria
Source: PLOS Glob Public Health. 2023 Dec 5;3(12):e0002667. doi: 10.1371/journal.pgph.0002667 (PMC10697517; doi:10.1371/journal.pgph.0002667)
Supplement: Attachment — Submitted filename: Point by point response file_August 2023.docx [file pgph.0002667.s003.docx]

| **Section** | **Editor/Reviewers’ comments** | **Author’s response** |
| --- | --- | --- |
| **Reviewer #2** | | |
| General comments | Overall, the authors have made some efforts to address the issues raised in the initial review. However, I still have some concerns about the objective of the study and the connection between the findings and conclusion. For example, how can the following findings be useful? “Being a farmer negatively predicted alcohol consumption”, “Compared to being in a single relationship, being married positively predicted alcohol consumption”, “Education and having at least a child were independent risk factors for contraceptive use but older age and being a farmer were protective”. This seems to imply that we should encourage people to be a farmer and not get married. Furthermore, how could the conclusion reflect the findings more concretely? | “Compared to being in a single relationship, being married positively predicted alcohol consumption” in the abstract section was a misinterpretation of the finding reported in the manuscript. See Table 3 and page 16, lines 238 – 239 in the main manuscript. This has been corrected: “Compared to being married, being in a single relationship positively predicted alcohol consumption”.  See pages 2 -3 lines 46 – 47.  The concern about the objective of the study and the connection between the findings and conclusion is noted. Summarily, we found an association between potential risk factors for cardiovascular diseases and demographic characteristics such as age, occupation, and marital status, but this relationship is not causal. To illustrate, farming is a predominant occupation in Benue state, and this reflects in the livelihood of her citizens. Thus, we chose farming as an important the variable of interest in our study design and analysis. Farming may discourage the consumption of alcohol, a potential risk factor for cardiovascular diseases and a lifestyle habit prevalent in the evening, a time when farmers are often tired from farming all day or may still be on the farm. To draw a connection between objective of the study, the findings, and the conclusion. We have added a sentence in the concluding section.  Older age, farming and being married are potential deterrents to lifestyle risk factors for cardiovascular diseases among this population.  See page 3 lines 50 – 51.  See page 16 lines 278 – 279. |
