# Supplementary material for: Potential risk factors for cardiovascular diseases and associated sociodemographic characteristics: A cross-sectional evaluation of a large cohort of women living with HIV in north-central Nigeria
Source: PLOS Glob Public Health. 2023 Dec 5;3(12):e0002667. doi: 10.1371/journal.pgph.0002667 (PMC10697517; doi:10.1371/journal.pgph.0002667)
Supplement: Attachment — Submitted filename: Point by point response file_Sept 2023.docx [file pgph.0002667.s004.docx]

| **Section** | **Editor/Reviewers’ comments** | **Author’s response** |
| --- | --- | --- |
| **Reviewer #2** | | |
| Major issues | |  |
| 1. Contradictory Results on Alcohol Consumption: The paper presents contradictory findings regarding the relationship between marital status and alcohol consumption. Line 173 states that being in a marital relationship increases the odds of alcohol consumption by 30%, while line 46 suggests that being single positively predicts alcohol consumption. This inconsistency needs to be addressed and clarified. | | The contradiction occurred as a result of a mistake in the wording of the text, which should have been “a non-marital relationship”. The word “marital relationship” has been replaced with “single relationship”.  Please see page 10, line 171 |
| 2. Unclear Relationship Between Contraceptive Use and Cardiovascular Risk: The paper briefly mentions that oral contraceptives predispose individuals to cardiovascular diseases (CVD) by causing thrombosis but the study lacks data on the number of subjects who have taken oral contraceptives as opposed to other measures. | | The statement on thrombosis is not relevant and has been removed. |
| 3. Data Selection and Potential Bias: The study includes 5,430 complete-case observations out of a total of 8,825, without providing a rationale for this selection or discussing the potential consequences of dropping the remaining observations. To address this issue, the authors should either justify the exclusion of these observations or consider techniques like data imputation or sensitivity analysis. | | The missingness in the data was completely at random. Thus, there is no risk of bias/systematic error. While imputation method is recognized for accounting missingness, this method is rather artificial (computer-generated responses) and would not be representative of exact responses from the study population. Finally, with over 5000 observations, we had sufficient power for any analysis. The statement “Of the 8,825 cohort, information on the outcome variables (alcohol consumption, smoking and contraceptive use) was incomplete for 3,395 observations” has been removed. |
| Minor issues | |  |
| 1. Unrelated Conclusion on Viral Suppression: The conclusion mentions the potential for lifestyle behavioral modifications to maintain viral suppression, a topic not covered in the study. This statement should either be removed or substantiated with relevant data or discussion. | | Thanks. Viral suppression has been removed from the conclusions.  See page 3, line 54  See page 17, line 275 |
| 2. Language Implying Causality: Although the study admits it cannot establish causal relationships, phrases like "Being a farmer negatively predicted alcohol consumption" imply causality. The language should be revised to reflect the correlational nature of the findings. | | The authors have replaced the word “predicted” or “predictor” with “ had a positive or negative association with” as necessary. See page 2, lines 43 – 45 and  page 3, line 47. |
| 3. Unclear Statement on p-value: The statement "A p-value of less than 0.05 was considered statistically significant, indicating the models were fit" is unclear. The authors should clarify what is meant by "the models were fit" in the context of a p-value less than 0.05. | | This was a mistake, and the sentence has been revised as “Statistical significance was considered at a p-value of less than 0.05”  See page 7, lines 132. |
| 4. Irrelevant Information on Tobacco Industry: The statement in line 209, "potentially, they are a target of the Tobacco industry," seems irrelevant to the study's focus and should be removed unless its relevance can be demonstrated. | | Thanks. The statement is irrelevant and has been removed |
| 5. Archaic Language: The use of archaic words like "thrice" is not recommended in scientific writing. Please consider using more contemporary terms. | | Thanks. The word “thrice” has been replaced with “three times”  See page 15, line 238 |
| Summary: The manuscript has several major and minor issues that need to be addressed before it can be considered for publication. Clarifying contradictory results, providing a rationale for data selection, and revising language to better align with the study's limitations are essential steps for improvement. | | The issues have been addressed. |
