# Supplementary material for: Potential risk factors for cardiovascular diseases and associated sociodemographic characteristics: A cross-sectional evaluation of a large cohort of women living with HIV in north-central Nigeria
Source: PLOS Glob Public Health. 2023 Dec 5;3(12):e0002667. doi: 10.1371/journal.pgph.0002667 (PMC10697517; doi:10.1371/journal.pgph.0002667)
Supplement: Attachment — Submitted filename: Response to reviewer comments.docx [file pgph.0002667.s001.docx]

| **Section** | **Editor/Reviewers’ comments** | **Author’s response** |
| --- | --- | --- |
| **Reviewer #1** | | |
| **Abstract** | | |
| Background | “Background: Age and education are known cardiovascular (CVD) risk factors in persons living with HIV (PLHIV).” This statement should be rewritten b/c when you say education is a known CVD risk factors it is not clear. Is it lack of information about CVDs? or having higher educational level, other? | Thanks. We meant lower educational level is a risk factor and this has been corrected in the abstract and introduction sections.  See page 2 line 29 - 30  See page 4 lines 67 – 68 |
| Methods | “We performed regression analyses of the influence of sociodemographic characteristics on potential cardiovascular risk factors, namely, smoking, alcohol consumption, contraceptive use.” Why you didn’t consider other CVDs risk factors like obesity (high cholesterol level/ higher BMI, sedentary life/lack of regular physical activity, hypertension and …………...? Suggestion: if you have no reason/s justification add it as limitation. | Studies have demonstrated potential cardiovascular risk factors persons living with HIV and AIDS including hypertension, hypercholesterolemia (seen in obesity) and dyslipidemia, as earlier stated in the introduction section of the manuscript (See page 4 lines 65). Thus, our goal was to explore other factors rather than repeat what is known. We have revised the abstract and introduction sections with justification.  “Males have a higher prevalence of cardiovascular (CVD) risk factors such as alcohol use, hypercholesterolemia, hypertension, obesity, and smoking based on limited data available from two tertiary health centers in Nigeria. Increasing age and lower educational level influence smoking among the same population in northeastern and northwestern Nigeria. Specifically in women living with HIV (WLHIV), the association between demographic characteristics and CVD risk factors has not been described”  See page 2 lines 27 – 32  See page 4 lines 63 – 72 |
| **Conclusion** | On conclusion “Targeting educated WLHIV who are smokers and alcohol users in single relationships has the potential to foster the maintenance of viral suppression, reduce CVD and improve treatment outcomes in WLHIV.” Is scientifically acceptable to keep smokers and alcoholics in single relationships instead of looking for another solution? Suggestion: look up any written guidelines/ articles and rewrite it. | Thanks. We should have written this clearly. Resources are often insufficient and there is always a need to prioritize based on available local data. Based on the findings of our study, we aimed to suggest the need to target smokers and alcohol users who are educated for behavioral modification of lifestyle factors. Essentially, reducing smoking and alcohol usage in WLHIV have the potential to reduce CVD. This has been rewritten.  “To improve HIV-related treatment efforts and outcomes, targeting smokers and alcohol users who are educated for lifestyle behavioral modification, has the potential to foster the maintenance of viral suppression and reduce cardiovascular disease risks.”  See page 2 lines 48 - 51 (abstract)  See page 16 lines 272 – 275 (main manuscript) |
| **Main manuscript** | | |
| **Introduction** | Paragraph 2 and 3 lack coherence and need edition for English language. | Paragraphs 2 and 3 were edited and merged.  See page 4 lines 63 – 75. |
| **Methods** | “This study was carried out between June and December 2017.” Write the specific date of data collection. | The study was carried out over a 7month period administering over 5000 semi-structured questionnaires. We prefer to retain it the way this was stated. Thanks. |
|  | Independent variables included age (18-39/≥40years), education (educated/none), occupation (farmers/non-farmers), and having children (≥1/none).” What is your evidence to categorize this predictor variables? cite it. | The influence of age (≥40 years) and childlessness and CVD risks and other health outcomes in have been included (See page 6, line 121, page 7 line 123). However, Benue state, is an agrarian state and is known as the food basket of the nation. We were not aware of studies which demonstrated the relationship between farming as a potential influence on CVD risk factors in persons living with HIV. Thus, we explored farming vs non-farming occupation as a potential risk factor in our study. |
| **Results** | Write table footnote for those “other” in the result table. Link result with table (cite result). | “other” comprised:  The footnote “other: fashion designers, hairdressers, health workers, housewives, midwives, public servants, students, and tailors” has been added to the result table.  See page 8 line 144.  The results have been linked with the table.  See page 7 line 139. |
|  | Clear explain which variables are analyzed by multivariate and what is the criteria to be analyzed by it after binary regression analysis i.e either indicate cut off P- value, or…... | We did not specify the cut-off for p-value for inclusion of the variables into multivariable analysis (not multivariate analysis). |
|  | The authors wrote, inferential statistics was obtained by computing bivariate /multivariate regression. Please write explicitly what type of regression (linear vs binary logistic) and model fitness/ procedures followed while you perform analysis. | Binary logistic regression was performed, and this correction has been made.  See page 7 line 123  We assessed the fitness of each of our models using the global null hypothesis test and the model was fit. A p-value of less than 0.05 was considered statistically significant.  See page 7 lines 126 - 127 |
| **Conclusions** | Conclusion should be written based on the finding. | This has been addressed. Thanks  See page 17 lines 271 – 275 |
